# Bioinformatic Analysis of Epigenetic and MicroRNA Mediated Regulation of Drought Responsive Genes in Rice

**DOI:** 10.1371/journal.pone.0049331

**Published:** 2012-11-08

**Authors:** Rafi Shaik, Wusirika Ramakrishna

**Affiliations:** Department of Biological Sciences, Michigan Technological University, Houghton, Michigan, United States of America; Max Planck Institute for Chemical Ecology, Germany

## Abstract

Drought stress response is a complex trait regulated at multiple levels. Changes in the epigenetic and miRNA regulatory landscape can dramatically alter the outcome of a stress response. However, little is known about the scope and extent of these regulatory factors on drought related cellular processes and functions. To this end, we selected a list of 5468 drought responsive genes (DRGs) of rice identified in multiple microarray studies and mapped the DNA methylation regions found in a genome wide methylcytosine immunoprecipitation and sequencing (mCIP-Seq) study to their genic and promoter regions, identified the chromatin remodeling genes and the genes that are targets of miRNAs. We found statistically significant enrichment of DNA methylation reads and miRNA target sequences in DRGs compared to a random set of genes. About 75% of the DRGs annotated to be involved in chromatin remodeling were downregulated. We found one-third of the DRGs are targeted by two-thirds of all known/predicted miRNAs in rice which include many transcription factors targeted by more than five miRNAs. Clustering analysis of the DRGs with epigenetic and miRNA features revealed, upregulated cluster was enriched in drought tolerance mechanisms while the downregulated cluster was enriched in drought resistance mechanisms evident by their unique gene ontologies (GOs), protein-protein interactions (PPIs), specific transcription factors, protein domains and metabolic pathways. Further, we analyzed the proteome of two weeks old young rice plants treated with a global demethylating agent, 5-azacytidine (5-azaC), subjected to drought stress and identified 56 protein spots that are differentially expressed. Out of the 56 spots, 35 were differently expressed in the sample with both demethylation and drought stress treatments and 28 (50%) were part of DRGs considered in the bioinformatic analysis.

## Introduction

In plants, epigenetic mechanisms including DNA methylation, histone modifications and certain small RNA (sRNA) mediated pathways regulate gene expression, chromatin structure and genome stability [1]. Dynamic epigenetic changes in response to endogenous and external stimuli play a definitive role in the plasticity of phenotype of an organism adapting to adverse environmental conditions. Thus, an increasing number of studies with the aid of high-throughput sequencing and genome tilling microarray technologies are focusing on exploring the role of epigenetic mechanisms in genome evolution and ecological adaptation. A recent study revealed the global cytosine methylation patterns in rice using methylcytosine immunoprecipitation (mCIP) combined with Illumina sequencing [2]. Genome-wide high resolution maps of DNase I hypersensitive (DH) sites from seedling and callus tissues of rice, which correlate with open chromatin structure revealed majority of DH sites to be located outside promoter regions and found 58% more DH sites in callus than in seedling [3]. Small RNAs (sRNAs) are increasingly found to regulate the epigenome through chromatin based pathways for gene silencing (RNA directed DNA methylation pathway), paramutation, genetic imprinting and epigenetic reprogramming [4]. A study of S-locus protein 11 genes (*SP11*) of *Brassica* demonstrated that sRNA derived from the dominant *SP11* allele trigger methylation of the promoter of recessive *SP11* gene [5]. While majority of sRNA in plants are small interfering RNAs (siRNAs) regulating transcriptional gene silencing, micro RNAs (miRNAs) play a key role in posttranscriptional gene silencing. Further, the distinction between siRNAs and miRNAs is becoming blurred, as both the molecules are intimately linked in terms of their origins and modes of operation [6]. Thus, integration and analysis of data on differential gene expression, epigenetic and sRNA mediated regulation would reveal a comprehensive picture of the dynamics of stress responsive genome in generating phenotypic diversity and could have significant implications in agriculture.

Rice is one of the most important economically important cereal crops accounting for about one-fifth of the total caloric intake of the human population worldwide [7]. Water deficit is a major abiotic factor affecting global crop yield and is known to induce a sequence of morphological, biochemical and molecular alterations that negatively affect plant growth and productivity [8]. With the advent of high-throughput technologies, dehydration tolerance in rice has been a subject of intense research resulting in a deluge of genomic, proteomic and metabolomic data [8], [9], [10], [11]. More than 5000 genes found to be differentially expressed in rice under drought stress by multiple studies were amalgamated by [11]. Many of these drought responsive genes (DRGs) are either poorly annotated or very little is known about their regulatory control especially through epigenetic and miRNA mediated mechanisms. So far a few studies analyzed the role of epigenetic mechanisms in drought response in rice. A study between drought-tolerant and drought-sensitive rice lines found a difference of about 12% in genome wide DNA methylation/demethylation and they also reported 70% of these changes revert back to original status while 30% remain even after recovery [12]. Another study in rice has shown the differential expression of DNA methyltransferases in different developmental stages, tissues and abiotic stresses contributing to *de novo* DNA methylation and maintenance [13]. A genome wide miRNA study identified 30 miRNAs that are differentially expressed in drought response [14].

In this study, we thematically collated and mapped the available information from different sources on DNA methylation; chromatin related proteins and sRNAs on DRGs and divided them into nine clusters based on presence/absence of these features and differential expression to pursue our goal of dissecting the orchestration of regulatory control in a plant cell responding to drought stress. Extensive characterization of the clusters based on a number of molecular features was performed. We also analyzed the proteome of young rice plants treated with 5-azacytidine (5-azaC) that causes global demethylation and grown in water deficit conditions to identify differentially expressed genes that are regulated by DNA methylation and play a role in drought response.

## Methods

### Drought responsive genes

The 5611 DRGs amalgamated by Ray et al. [11] from various drought studies on rice [11], [15], [16], [17] were selected for this analysis. The 112 genes with Affymetrix probe IDs mapping to *Oryza sativa* ssp *indica* were filtered out. The rest of the genes were matched with MSU release 7.0 of *Oryza sativa* ssp *japonica* (http://rice.plantbiology.msu.edu) and 31 obsolete loci from older MSU releases were also left out, leaving 5468 unambiguous DRGs belonging to *Oryza sativa* ssp *japonica* with latest annotation (Table S1). A random list of 5000 genes was generated using a Perl script from MSU7 annotation from all rice genes excluding pseudogenes and retrotransposon related genes. The 835 (15% of 5468) DRGs in the list were retained to truly account for randomness.

### Epigenetic features

The mCIP-seq or DNA methylation reads in rice [2] were mapped on to the genomic location of the DRGs using a Perl script. The reads localized between transcriptional start site (TSS) and end of each gene with an overlap cut-off value of minimum 50 bases were collated and classified as genic DNA methylation reads and those falling 1 kb upstream region of the TSS were collated and classified as promoter DNA methylation reads. The genes annotated as chromatin-associated proteins (CAPs) by the chromatin database (ChromDB) [18] among the DRGs were identified. The plant miRNA database (PMRD) [19] has 2641 miRNAs of rice (both experimental and computationally predicted miRNAs, including all the miRNAs reported in the miRBase database [20]) and their target genes predicted by psRNATarget server [21]. One or more micro RNAs (miRNAs) targeting each of DRGs as reported in plant microRNA database (PMRD) were identified.

### Classification of DRGs into clusters

**Figure 1:**
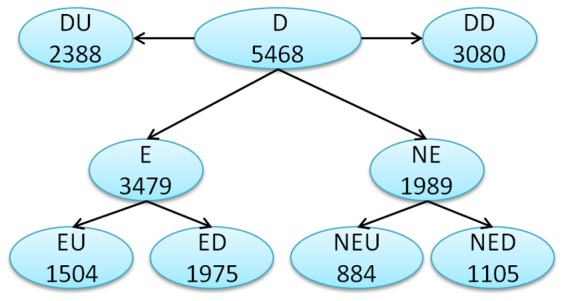
Classification of Drought Responsive Genes (DRGs) into nine clusters based on epigenetic/miRNA features and differential expression. Cluster D: all DRGs, DU: upregulated DRGs, DD: downregulated DRGs, E: Genes with any or all epigenetic/miRNA features, NE: no epigenetic/miRNA features, EU: E with upregulated DRGs only, ED: E with downregulated DRGs only, NEU: NE with upregulated DRGs only and NED: NE with downregulated DRGs only.

The DRGs were classified into nine clusters as shown in figure 1. All of the 5468 considered as part of the cluster D (Drought). The DRGs with any of the following features: DNA methylation reads overlapping promoter region or genic region, miRNA target and ChromDB gene were grouped together and classified as cluster E (epigenetic and miRNA) and those without any of the above features were classified as cluster NE (non epigenetic and miRNA). Each of the D, E and NE clusters were further classified into DU (drought upregulated) and DD (drought downregulated), EU (with epigenetic and miRNA features and upregulated) and ED (with epigenetic and miRNA features and downregulated), NEU (without epigenetic and miRNA features and upregulated) and NED (without epigenetic and miRNA features and downregulated) to reflect up or downregulation of gene expression.

### Gene ontology analysis

The genes in each of the clusters were analyzed using the Singular Enrichment Analysis (SEA) tool by agriGO [22] at default settings of Fisher t-test (p<0.05), False Discovery Rate (FDR) correction by Benjamini-Yekutieli method and five minimum number of mapping entries against species specific pre-computed background reference.

### Proteome analysis

The predicted protein-protein interactions (PPIs) shown by the protein(s) coded by every gene with all other protein(s) within the cluster were identified using the Search Tool for the Retrieval of Interacting Genes/Proteins database (String-DB) [23] with a combined score p-value<0.04.

The gene IDs annotated as members of different transcription factor (TF) families by plant transcription factor database (PlnTFDB v3.0, http://plntfdb.bio.uni-potsdam.de/v3.0/) [24] were searched against the IDs of all DRGs. The plnTFDB had 3119 protein models belonging to 2399 genes annotated as TFs and were arranged in 80 families (TF families and other transcriptional regulators) for the species *Oryza sativa* subsp. *japonica*. Each of the TF family was analyzed to find the clusters they are enriched in. The lists of overlapping TF families in different clusters were analyzed using the tool Venny [25].

The protein domains present in all of the DRGs based on the classification by provided Pfam [26] were obtained from http://rice.plantbiology.msu.edu/ and were analyzed for overrepresented protein domains. Further, each of the domain family was analyzed to find the distribution of its members in the nine clusters.

The information about the metabolic pathway-associated genes was obtained from the data provided in RiceCyc [27]. Each pathway was analyzed to find the number of genes present in each of the clusters and the percentage of DRGs over total number of genes in that pathway.

### Drought stress and 5-azaC treatments

The protocol was adapted and modified from Boyko et al. [28]. The seeds of *Oryza sativa* ssp *japonica* cultivar Nipponbare obtained from the National Plant Germplasm System (NPGS) of the United States Department of Agriculture - Agricultural Research Service (USDA-ARS) were sterilized and germinated in a sterile petri plate wetted with half-Murashige and Skoog (MS) medium and grown in dark for 4 days at room temperature. Twenty young seedlings were transferred to magenta box each with 50 ml of half-MS medium for control plants and 50 ml of half-MS medium and 1–50 µM 5-azaC for treated plants (Thermo Fisher Scientific, NJ) and grown for two weeks in the dark at 28°C-day/25°C-night temperature, 12-h-light/12-h-dark cycle, and 50% humidity. Drought stress was given for 5 hrs according to Dai et al. [29] by transferring the young plants to Whatman 3 MM paper in a sterile petri dish.

### Two dimensional SDS-PAGE, in-gel digestion and MALDI-TOF

Total protein from four groups (control (C), drought stress (DS), 10 µM 5-azaC (A) and 10 µM 5-azaC with drought stress (ADS)) was isolated using ReadyPrep Protein Extraction Kit (Bio-Rad, CA) and quantified using BCA Assay. About 150 µg of protein sample from each group was incubated in 200 µl of rehydration buffer (8 M urea, 2 M thiourea, 2% CHAPS and 50 mM DTT). Isoelectric focusing was carried out using 11 cm immobiline dry strips (Bio-Rad, CA) with a non-linear pH 3–10 gradient. Strips were rehydrated using programmed voltage gradients at 20°C for a total of 12 kVh and separated for 1 h at 500 V, 1 h at 1000 V, 2 hrs at 6000 V and 40 min at 6000 V. The IPG strips were reduced in equilibration buffer-I (0.375 M Tris-HCl, pH 8.8, 6 M urea, 20% glycerol, 2% SDS, and 50 mM DTT) for 20 min at 25°C and alkylated for 20 min in equilibration buffer-II containing 150 mM iodoacetamide. The equilibrated strips were placed on top of 15% polyacrylamide gels and run for 2.5 hrs at 100 V. Proteins were visualized by Coomassie Imperial Protein Stain (Pierce, Rockford, IL). Differentially expressed proteins between all groups were identified using ImageMaster (GE Healthcare Biosciences, PA).

Protein spots from 2D electrophoresis were excised from gels based on their fold change (>2-fold) and resolution. The gel pieces were destained twice with 200 µl of 50% acetonitrile (MeCN)/25 mM NH_4_HCO_3_ buffer (pH 8.0) at room temperature for 20 min, washed once with 200 µl of 100% MeCN and vacuum dried by a speedvac concentrator (Savant, Holbrook, NY). The gel pieces were rehydrated with 13 ng/µl sequencing grade modified trypsin (Promega, Madison, WI) in 25 mM NH_4_HCO_3_ and incubated at 37°C overnight. Peptides were subsequently extracted twice with 50 µl of 50% MeCN/5% formic acid for 15 min at 37°C. All extracts were combined and dried. The peptides were eluted with 5 µl of 75% MeCN/0.1% TFA. The peptides were analyzed using matrix assisted laser desorption/ionization time of-flight mass spectrometry (MALDI-TOF MS) (Microflex, Bruker). About 0.5 µl of 2,5-dihydroxybenzoic acid (DHB) matrix was loaded on a 96well ground steel MALDI plate followed by 0.5 µl of peptide extract. Each sample was scanned with 1000 laser shots at 60% laser strength. The mass spectra were corrected for background subtraction and mass calibration. Protein masses were identified by searching NCBI_nr database through MASCOT search engine with 1 missed cleavage, ±100 ppm of mass tolerance, carbamidomethylation of cysteines as fixed modification and oxidation of methionine as variable modification. To identify the MSU7 IDs of the homologous proteins, BLASTP searches were performed (http://rice.plantbiology.msu.edu/) and the best hits were selected.

## Results

### Epigenetic features of DRGs

**Figure 2:**
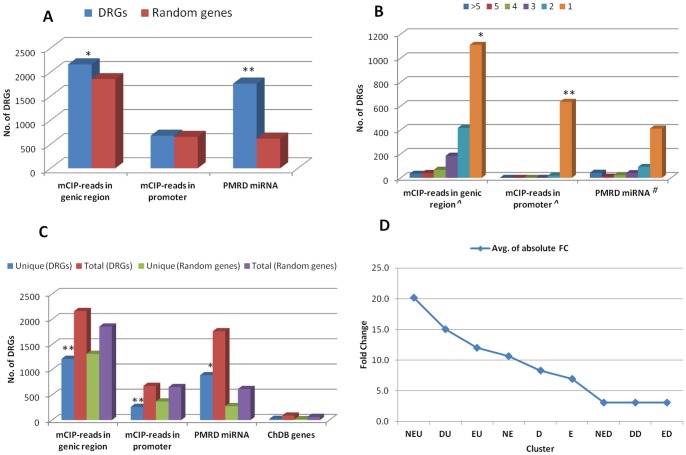
Enrichment of epigenetic features in DRGs. A) Epigenetic features of DRGs versus random set. The number of mCIP-reads mapped to the genic region of DRGs compared to the random set. In the same way mCIP-reads that mapped to promoter region were compared. Total numbers of miRNAs from PMRD database targeting the DRGs were compared to the random set. B) Distribution of multiple instances of epigenetic features on DRGs. ∧Each of the bars represents number of DRGs mapped with given number of mCIP-reads only. #Each of the bars represents number of DRGs targeted by given number of miRNA only. C) Comparison of sets of genes with unique epigenetic features in DRGs with the random set. Unique represents the set of genes with only one of the three epigenetic/miRNA features and all represents number of genes with a particular feature and with one or more other epigenetic/miRNA features. D) Distribution of the average of absolute fold change of gene expression from [11] for the nine clusters. * indicates significant Z-score at p<0.05 and ** indicates significant Z-score at p<0.01.

A total of 2162 DRGs (39.5%) with one or more methylation reads (3633 total reads) falling in genic regions were identified (Fig. 2A), which is statistically significant (z-score: 2.58 at p<0.05) compared to a set of 5000 random genes. About 853 DRGs (40% of 2162) had more than one methylation read mapped to their genic region (Fig. 2B). The average gene length of the DRGs was 3522 bases while that of all genes in rice was 6656 (including transposon element (TE) genes). The average gene length of the DRGs with at least one methylation read in their genic regions was 4725 bases and those without any methylation reads in their genic regions was 2735 bases. Our finding of significantly smaller average length of genes without any methylation reads (57% reduction) specifically among DRGs suggests a correlation between methylation and their gene size. Out of the 2162 DRGs, 461 (21.3%) had one or more methylation reads in the first 1000 bases from TSS. We identified 1249 (22%) and 913 genes (16.6%) with methylation reads in their genic region that were down and upregulated in drought stress, respectively. We found 678 DRGs (12.3%) with one or two mCIP-reads mapped to their promoter regions (Fig. 2A). Out of 678, 213 had methylation reads in the first 200 bases upstream of TSS. Interestingly, 296 (43%) DRGs with methylation reads mapped to their promoter region also had at least one methylation read mapped on to their genic region.

In total, 1761 DRGs (32% ) were potential targets of one or more miRNAs which is highly significant compared to the random set (616 or 12%) with a z-score of 24.25 (p<0.01) (Fig. 2A). A number of DRGs were predicted to be targets of multiple miRNAs (Fig. 2B). Ninety one DRGs were predicted to be targets of 10 or more miRNAs (Table S2A). Three DRGs (LOC_Os08g13430 (expressed protein), LOC_Os05g18294 (SEC14 cytosolic factor family protein) and LOC_Os11g25780 (PB1 domain containing protein)) had more than 150 miRNAs targeting them. Out of 2641 miRNAs in PMRD, 1771 (67%) had at least one DRG as target with 82 miRNAs predicted to target 10 or more DRGs (Table S2B). The regulation of about one-third (32%) of DRGs by two-thirds (67%) of all known/predicted miRNAs reemphasizes the importance of miRNA mediated regulation of these DRGs and the need to comprehensively understand their mechanism of action. The miRNAs, osa-miRf10273-akr predicted by miMatcher pipeline [30] and osa-miR414 experimentally identified in the moss *Physcomitrella patens* [31], were predicted to target highest number of DRGs (103 and 75, respectively). We found 88 DRGs (17% of 514 rice genes in ChromDB) that are chromatin related genes. Interestingly, 66 of these 88 DRGs (75%) were downregulated suggesting that the chromatin landscape of the rice genome has been dramatically altered in drought response.

The DRGs with only one of the three epigenetic features studied were analyzed and compared to a random set of 5000 genes (Fig. 2C). The DRGs with DNA methylation in either genic or promoter region seem more likely to share other epigenetic features. This is evident by the significant negative z-score of −9.5 and −6.4 (p<0.01) for the number of DRGs with only DNA methylation in genic region and only DNA methylation in promoter region, respectively as the epigenetic feature compared to the random set. The number of DRGs targeted by miRNA exclusive of other epigenetic features was 890 (16% of all DRGs) while that for random set was 276 (∼5%) (z-score 2.45 p<0.05). The number of ChromDB genes exclusive of other features was 25 while that of random set is 18. The number of DRGs with DNA methylation in genic region which are also targets of miRNA are 736 (13% of all DRGs) while the same for random set is 299 (5%) (z-score 12.8 at p<0.01). Similarly, number of DRGs with DNA methylation in promoter region which are also targets of miRNA are 219 (4%) while the same for control set is 63 (1.2%) (z-score 8.6 at p<0.01). The number of DRGs having DNA methylation in genic and promoter regions and also are targets of miRNA (PMRD) (all three epigenetic features) are 104 (1.9%) while the number in random set is 26 (0.5%) (z-score 6.3 at p<0.01).

### Cluster analysis of DRGs elucidates different gene expression patterns

Overall, cluster E had 63.6% of all DRGs and the clusters with genes that are downregulated had higher number of genes even upon classifying into sub clusters (Clusters DD, ED, NED compared to DU, EU and NEU) (Fig. 1). Comparison of average of the absolute fold change of gene expression of each of the clusters showed a clear trend of higher fold change for all the clusters with upregulated genes (EU, DU and NEU) and lower fold change for all the clusters with downregulated genes (ED, DD and NED) (Fig. 2D). The positioning of the cluster NEU at top as shown in figure 2D, suggests that the genes in cluster NEU could be expressed through a simpler route as they are not under direct control of epigenetic and miRNA mediated mechanisms. On the other hand, the lowest average fold change of gene expression of cluster ED could possibly be due to tighter control of the genes in this cluster and are very selectively expressed, specifically in stress response.

### GO analyses of the clusters reveal a number of novel biological processes and functions of DRGs

In total, we found 1011 significant GOs (p<0.05) for all of the nine clusters combined. These comprised 320 unique GOs out of which 189, 90 and 41 were related to biological process (BP), molecular function (MF) and cellular component (CC), respectively. Out of these 73 GOs (22.9%) are unique to only one of the 9 clusters (Table S3). Besides reporting most of the GOs that are known to be enriched in DRGs by other studies, our analysis revealed a vast number of novel GOs as a result of clustering based on the underlying regulatory information. For instance, the GO “response to biotic stimulus” was found to be significant (p = 0.00026) only in cluster D. Even upon classifying the cluster D into clusters DD and DU this term was not significant. Conversely, the GO “ncRNA metabolic process” was found to be significant (p = 0.0021) only in cluster ED and was not significant in other clusters including cluster D.

**Figure 3:**
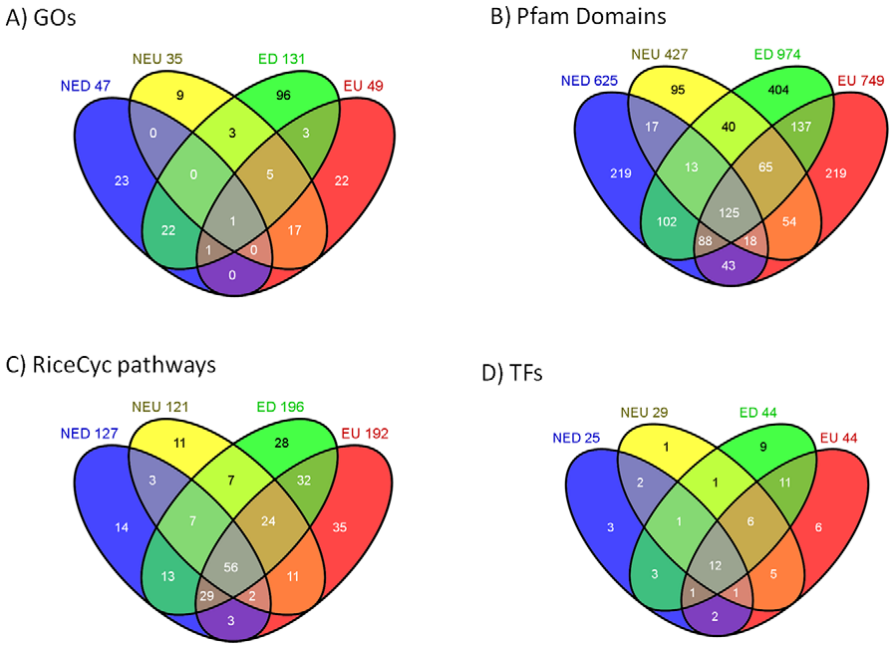
Four way venn diagrams depicting overlap of different characteristics between the clusters NED, NEU, ED and EU. A) GO terms analyzed by AgriGO, B) Protein domain families as per Pfam database, C) Metabolic pathways by RiceCyc and D) Types of transcription factors as reported by PlnTFDB.

Each of the clusters revealed distinct GOs that clearly define their properties. Significant overlapping GOs were observed in the groups that are either up or downregulated such as 22 common and exclusive GOs between NED and ED and 17 between NEU and EU (Fig. 3A and Table S4). On the other hand, there were no shared GOs common and exclusive between NED and NEU and only 3 GOs between ED and EU. About 73% of GOs of the ED are unique to ED (overrepresented) and a major portion of the remaining GOs were shared with cluster NED exclusively. A number of GOs that are unique to NED are related to photosynthesis such as “photosystem”, “photosynthetic membrane”, “photosynthesis light harvesting” and other terms include “structural molecule activity”, “protein folding”, and “response to oxidative stress”. Conservation of energy by reduction of photosynthetic activity and translation are known drought response mechanisms. Exclusive enrichment of these processes in the cluster NED suggests they are probably not under direct epigenetic and miRNA control.

Cluster NEU shows a peculiar behavior of not overlapping with NED with no common GOs in 3 out of 4 possible combinations which suggests the clear demarcation of processes controlled by genes that belong to NEU and NED (Fig. 3A). Out of the 14 common GO BP terms between clusters NEU and EU, 11 are related to regulatory processes (Table S4). The GOs “response to water” (p<0.00009) and “response to abiotic stimulus” (p<0.0007) were also common to NEU and EU. The GOs unique to cluster NEU are mostly related to “RNA biosynthesis”, “metabolism”, “transcription” and “regulation of these processes” (Fig. S1). This result is in agreement with the expectation that genes involved in processes like RNA biosynthesis and transcription perform basic housekeeping functions of the cell and do not require subtle control by higher order regulatory mechanisms. Yet, upregulation of genes with these functions suggest requirement of the cell under stress to produce a large quantities of different kinds of RNAs as part of drought response.

**Figure 4:**
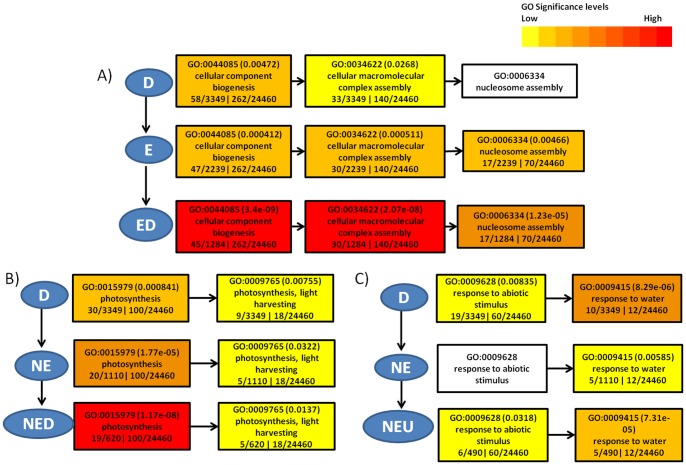
Examples of enrichment/depletion of significant GO terms in DRG clusters. A) and B) show increase and C) shows decrease in the significance of GO terms as we move to sub clusters indicated by vertical arrows. Also shown are the changes in significance of GO terms from a parent to child GO term indicated by horizontal arrows. White boxes denote GO terms that are not significant (p>0.05).

The GOs that are unique to the EU overall seem to be related to protein modification processes especially “serine/threonine phosphatase activity” which is enriched significantly (p = 8.00E-08) in addition to “signal transduction processes” and “response to osmotic stress”. Reduction of transpiration by stomatal closure and accumulation of osmoprotectants in response to the resulting osmotic stress are well known mechanisms of drought response. Cluster ED with highest number of significant GOs is also the cluster with highest number of non-overlapping GOs (96/131 GO terms or 73%). This cluster shows a high number of terms related to nucleosome and cytoskeletal reorganization, and metabolic processes implying the complex regulation of energy conservation mechanisms by downregulation of a number of metabolic processes and reorganization of a number of cellular structures inside the cell responding to drought. A few examples showing enrichment/depletion of GOs in DRGs due to clustering are illustrated in figure 4.

### Clusters EU, ED, NEU and NED exhibit distinct patterns of protein-protein interactions

**Figure 5:**
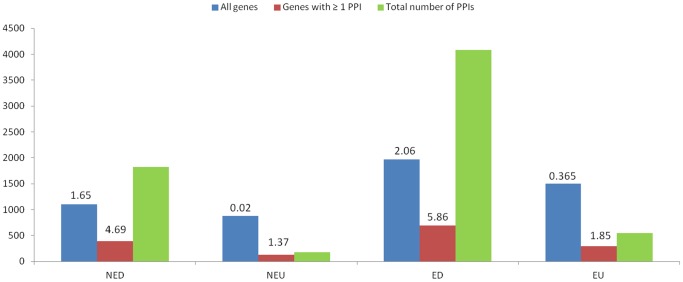
Number of protein protein interactions (PPIs) found in the four leaf clusters. The numbers above the bars represent average number of PPIs per gene over total number of PPIs found in the cluster and average number of PPIs per gene among genes with ≥1 PPI over total number of PPIs found in the cluster.

**Figure 6:**
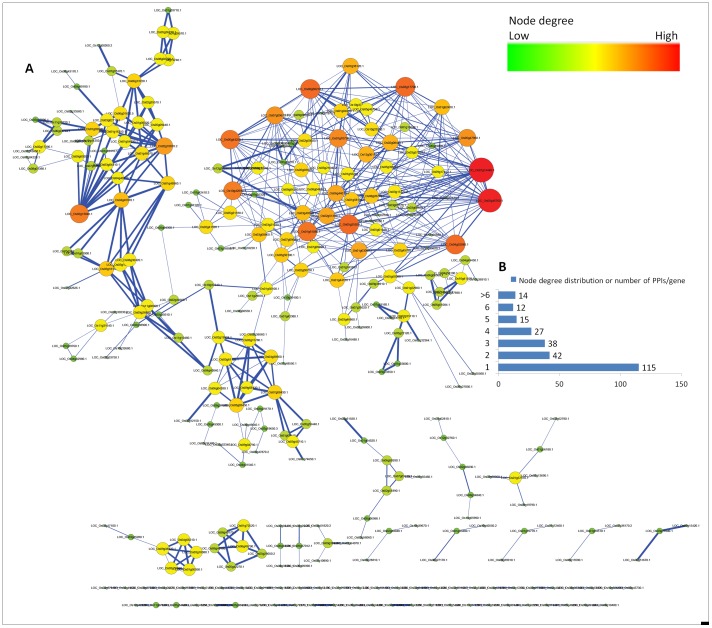
Protein-protein interactions (PPIs) network of cluster-EU. A) Network diagram showing DRGs as circles with size and color corresponding to number of PPIs. The large size of the circle and color intensity indicate higher number of PPIs,. Thickness of edges between two nodes is based on String-DB score. Thicker edges denote high String-DB scores, B) The number of genes with different number of PPIs (X-axis).

The STRING database analyses revealed higher number of interactions in downregulated clusters with 4.69 and 5.86 PPIs per gene with at least one PPI in NED and ED, respectively, compared to the upregulated clusters with 1.37 and 1.85 PPIs per gene with at least one PPI in NEU and EU, respectively (Fig. 5). About 35% of genes show PPIs in clusters NED and ED while only about 14.5% and 19.6% of genes show PPIs in clusters NEU and EU, respectively. This suggests the probable location of a number of down regulated genes at the bottom of regulatory cascades as evident by their significant GOs related to multitude of processes including metabolic, biosynthetic, and photosynthetic processes which involve many PPIs for the synthesis or degradation of a number of metabolites/biological substances and upregulated genes at the top of regulatory cascades controlling a few critical reactions as supported by the fact that 11 out of 14 GO BPs common and exclusive to EU and NEU are related to regulatory processes and they also show high average of the absolute fold change of gene expression. A list of top ten DRGs with highest number of PPIs in the clusters EU, ED, NEU and NED are given in table 1. The top ten DRGs of EU contain three TFs, three kinase family genes and a jmjC domain coding gene which regulates chromatin reorganization processes [32] suggesting that these genes are major players in drought response. The complete PPI network of EU is shown in figure 6 and the individual PPIs along with their String-DB scores are given in table S5. Out of the 295 DRGs in EU, 115 had only one PPI and 14 had >6 PPIs (Fig. 6B). Two DRGs, LOC_Os01g14440 (OsWRKY1v2 - superfamily of TFs having WRKY and zinc finger domains) and LOC_Os05g46760 (STE_MEKK_ste11_MAP3K.19- STE kinase, part of the MAPK signaling cascade) had 28 and 26 PPIs each with other members in EU. Both the genes have one DNA methylation read overlapping with their genic regions and LOC_Os05g46760 is also predicted as a target of osa-miRf12002-akr.

**Table 1:** Top 10 DRGs of clusters ED, EU, NED and NEU based on number of PPIs.

MSU ID	PPI Count	Gene Description	DNA methylation and miRNA features*
**Cluster ED**			
LOC_Os07g32880	84	ATP synthase gamma chain, putative, expressed	chr7_solexa13_1006351 (p)
LOC_Os10g10180	80	methyltransferase domain containing protein, putative, expressed	osa0miRf118720akr
LOC_Os01g03040	80	expressed protein	chr1_solexa12_1000315;chr1_solexa12_1000316; (g)
LOC_Os04g41340	78	4-nitrophenylphosphatase, putative, expressed	osa0miRf108630akr
LOC_Os08g06530	75	rubredoxin family protein, putative, expressed	chr8_solexa13_1000861 (g)
LOC_Os12g38640	75	expressed protein	chr12_solexa13_1007345 (g)
LOC_Os07g07540	74	SHOOT1 protein, putative, expressed	osa0miRf102730akr;osa0miRf109470akr
LOC_Os08g07060	73	CRR6, putative, expressed	chr8_solexa13_1000916 (g); osa0miRf115530akr
LOC_Os02g01150	73	erythronate-4-phosphate dehydrogenase domain containing protein, expressed	osa0miRf118380akr
LOC_Os02g47020	71	phosphoribulokinase/Uridine kinase family protein, expressed	chr2_solexa13_1008132;chr2_solexa13_1008133 (g)
**Cluster EU**			
LOC_Os01g14440	28	WRKY1, expressed	chr1_solexa12_1002043 (g)
LOC_Os05g46760	26	STE_MEKK_ste11_MAP3K.19 - STE kinases include homologs to sterile 7, sterile 11 and sterile 20 from yeast, expressed	chr5_solexa13_1007768 (g); osa-miRf12002-akr
LOC_Os05g25920	18	expressed protein	chr5_solexa13_1004521 (p); osa-miRf10947-akr
LOC_Os03g17700	18	CGMC_MAPKCGMC_2_ERK.2 - CGMC includes CDA, MAPK, GSK3, and CLKC kinases, expressed	chr3_solexa12_1002251 (g)
LOC_Os08g38210	18	transcription factor BIM2, putative, expressed	chr8_solexa13_1007354 (g)
LOC_Os04g52840	18	tyrosine protein kinase domain containing protein, putative, expressed	chr4_solexa13_1009408 (g)
LOC_Os06g44250	17	haemolysin-III, putative, expressed	osa-miRf12029-akr
LOC_Os01g61080	17	WRKY24, expressed	osa-miRf10947-akr
LOC_Os10g42690	16	jmjC domain containing protein, expressed	JMJ706 (ChromDB ID); osa-miRf10002-akr
LOC_Os02g13840	16	citrate synthase, putative, expressed	chr2_solexa13_1001684 (p)
**Cluster NED**			
LOC_Os04g51792	72	PAP fibrillin family domain containing protein, expressed	N.A
LOC_Os02g42570	69	ferredoxin-thioredoxin reductase, variable chain, putative, expressed	N.A
LOC_Os01g68450	67	expressed protein	N.A
LOC_Os03g17070	63	ATP synthase B chain, chloroplast precursor, putative, expressed	N.A
LOC_Os03g16050	62	fructose-1,6-bisphosphatase, putative, expressed	N.A
LOC_Os10g15300	60	expressed protein	N.A
LOC_Os08g27010	59	APE1, putative, expressed	N.A
LOC_Os01g55570	58	expressed protein	N.A
LOC_Os02g51820	57	expressed protein	N.A
LOC_Os07g13969	55	expressed protein	N.A
**Cluster NEU**			
LOC_Os01g64470	13	harpin-induced protein 1 domain containing protein, expressed	N.A
LOC_Os01g72530	12	OsCML31 - Calmodulin-related calcium sensor protein, expressed	N.A
LOC_Os06g04240	12	expressed protein	N.A
LOC_Os06g10210	11	expressed protein	N.A
LOC_Os03g53020	9	helix-loop-helix DNA-binding domain containing protein, expressed	N.A
LOC_Os10g25290	9	ZIM domain containing protein, putative, expressed	N.A
LOC_Os06g46950	8	EF hand family protein, putative, expressed	N.A
LOC_Os11g10470	8	expressed protein	N.A
LOC_Os04g43680	7	MYB family transcription factor, putative, expressed	N.A
LOC_Os03g60570	7	ZOS3-22 - C2H2 zinc finger protein, expressed	N.A

* g and p in brackets denote that the methylation read(s) overlap genic and promoter regions respectively. N.A indicates not applicable.

### Characterizing the DRG clusters based on transcription factor family distribution

Out of the 5468 DRGs, 450 (8%) were annotated as transcription factor genes (Table 2). Interestingly, these 450 Drought Responsive Transcription Factors (DRTFs) represent most of the TF families (64/80 families in PlnTFDB) (Table S6). Although the cluster size of DU was smaller than DD, higher numbers of DRTFs were present in DU. Similarly NEU had the highest percent of TFs even though it had the least number of genes among all the nine clusters and NED had the least percent of TF genes. These results are similar to the trends observed in our fold change analysis (Fig. 2D).

**Table 2:** Number of transcription factor genes in the nine clusters.

Cluster	No. of genes	No. of TF genes	Percent of TF genes in the group
**Rice genome**	55986	2399	4.28
**D**	5468	450	8.23
**NE**	1989	148	7.44
**E**	3479	302	8.68
**NED**	1105	45	4.07
**NEU**	884	103	11.65
**ED**	1975	144	7.29
**EU**	1504	158	10.51
**DD**	3080	190	6.17
**DU**	2388	261	10.93

Majority of the members of important TF families AP2-EREBP (29 out of 38 DRTF genes), bHLH (20/32), bZIP (19/27), C3H (9/9), DBP (3/3), HSF (9/10), LOB (5/6), NAC (22/30), PHD (6/7), Tify (6/7) and Trihelix (5/5) were in cluster DU while majority of the members of TF families CCAAT (7/9), G2-like (9/11) and MADS (14/18) were in ED (Table S6). The number of TF families that are unique and common between different leaf clusters is shown in figure 3D. AP2-EREBP is one of the largest TF families unique to plants and is characterized by the presence of AP2 DNA-binding domain. AP2-EREBP has the highest number of DRTFs and majority of the members (75%) are upregulated in drought response suggesting upregulation of a number of functional roles attributed to this family. Similar trend was shown by other large TF families, namely NAC and bZIP. A number of major TF families were exclusively found in EU and ED (MADS, C2C2-CO-like, CCAAT, and HMG). Many TF families show bias to one of the four clusters. For example, 12/21 WRKY DRTFs are present in EU and 14/18 MADS DRTFs are present in ED. One of the major role played by MADS box genes is development of plant reproductive structures, specifically floral meristem and organ identity [33]. The enrichment of MADS TFs in ED suggests that these mechanisms are subtly controlled and downregulated as part of drought resistance to conserve energy.

### Protein domain family distribution analysis reveals enrichment of major domain families in clusters with epigenetic and miRNA features

Rice genome has 33779 genes with at least one Pfam domain belonging to 3337 families. Out of 5468 DRGs, 4348 have Pfam domains belonging to 1639 families (Table 3) suggesting the wide range of changes involved in drought response. Overall the clusters E and DU show significantly higher percentage of genes with at least one Pfam domain compared to NE and DD (cluster-E 82.5%, G-statistic-51.4 and DU 79.6%, G-statistic-13.1 compared to NE 74.2% and DD 75.5% respectively). Figure 3B shows the number of domain families that are unique and common across different combinations of clusters. The trends observed here are similar to those in figure 3A with NED and ED, NEU and EU, and ED and EU showing higher overlap than other cluster combinations.

**Table 3:** Number of genes with pFAM domains and the number of different domain families found in the nine clusters.

Cluster	No. of genes	No. of genes with one or more Pfam domains	No. of domain families
**Rice genome**	55986	33779	3337
**D**	5468	4348	1639
**NE**	1989	1477	879
**E**	3479	2871	1308
**NED**	1105	829	625
**NEU**	884	648	427
**ED**	1975	1617	974
**EU**	1504	1254	749
**DD**	3238	2446	1271
**DU**	2388	1902	915

A number of major domain families were enriched in cluster E suggesting many proteins with functional roles in signal transduction and metabolism are under direct epigenetic control. For example 182/1144 Pkinase domains in rice are found in DRGs out of which 138 or 75% were present in cluster E (Table S7). The other domains showing similar trend in cluster E include LRR_1 (63/87 domains found in all DRGs), NB_ARC (23/32), SRF-TF (18/18), peptidase_S10 (14/15) and terpene_synth (11/13). Further, all of the above mentioned domains were significantly enriched in cluster ED suggesting the processes controlled by these domains are highly downregulated in drought response. Many other domains that were enriched in cluster E were further enriched in cluster EU. Examples include zf-C3HC4 (40/63 domains in all of DRGs were part of E out of which 26 were part of EU), PP2C (20/29 E and 17/29 EU) and raffinose_syn (5/5 E and 4/5 EU). Raffinose family oligosaccharides (RFO) were found to act as ROS scavengers and also play a role in protection against freezing, desiccation and high temperature stress [34]. All seven dehydrin domain containing genes found in the rice are part of the DRGs considered in this study and all of the seven were upregulated. Dehydrin domain containing proteins are produced in plants in response to low temperatures and drought stress and protect membranes from damage [35].

A number of DUFs (Domain of Unknown Function) also showed enrichment in distinct clusters, suggesting that these domains could be playing an important role in drought response that is unknown. For example, 8/11 DUF221 domains were part of DRGs out of which 7 were part of DU and 5 were part of EU. The only annotation available for DUF221 is that it is a family of hypothetical transmembrane proteins (http://Pfam.sanger.ac.uk/family/PF02714). A number of domains and families although present in high numbers in rice, were found to be underrepresented in DRGs including zf-CCHC, hATC, chromo, Peptidase_C48 and FAR1 domains (Table S7).

### Metabolic pathway analysis reveals enrichment of pathways involved in synthesis of a number of amino acids, peptides and sugars in cluster EU which function as osmoprotectants and antioxidants

We found 275 out of 357 pathways listed in RiceCyc to be differentially regulated in drought stress (Table S8). The distribution of the pathways in different DRG clusters is shown in table S9. About 20% of 275 pathways were common to all of the four leaf clusters NED, NEU, ED and EU (Fig. 3C). Approximately 35% of the pathways are exclusive to cluster E while only 10% are unique to NE. DRGs involved in amino acid synthesis pathways including proline, alanine, citrulline, methionine were significantly enriched in cluster EU (Table S8), which are known to serve as osmoprotectants and antioxidants as part of drought response. [36], [37]. Glutathione (GSH) is a tripeptide known to act as a redox sensor for environmental stress. Antioxidant defense reactions, which use GSH as an electron donor for the regeneration of ascorbate are considered as the main pathway of superoxide and H_2_O_2_ removal [38]. We found gamma-glutamyl cycle and ascorbate biosynthesis pathways to be enriched in the cluster EU. Trehalose functions as a stress protectant, stabilizing proteins and membranes against destruction [39]. Multiple genetic studies have proposed trehalose pathway as a central metabolic regulator [40]. There are 19 genes linked to trehalose biosynthesis I pathway in RiceCyc, out of which 7 are part of the DRGs. We found all of the 7 DRGs to be part of DU and 5 to be part of EU. The DRGs encoded for the enzymes involved in GDP-D-rhamnose and GDP-L-fucose synthesis which are components of primary cell wall were also found to be significantly enriched in EU. Jasmonic acid is a hormone known to induce lipoxygenases that protect against membrane alterations during water stress [41]. Twelve out of 13 DRGs found to be involved in jasmonic acid biosynthesis pathway were under epigenetic control and eight of those were enriched in cluster EU.

A number of biosynthetic pathways were found to be specifically enriched in ED including those related to fatty acids, nucleotides, sugars like sucrose and UDP-D-xylose, cellulose, heme, lysine, phenylpropanoid and folate derivatives. While the degradation pathways of amino acids like tryptophan and valine were enriched in ED, their biosynthetic pathways were enriched in EU. All eight of the DRGs involved in tRNA charging pathway were part of DD and 7 of which were also part of ED. Out of the 26 genes involved in photorespiration, 10 are DRGs and 7 of which were part of ED. A number of basic metabolic pathways were significantly enriched in cluster E but dispersed between the clusters EU and ED including biosynthesis and degradation pathways of glucose, galactose and starch, TCA cycle, biosynthesis of phospholipids, lipoxygenases (LOX), brassinosteroids, cysteine, methionine and degradation pathway of sucrose.

### Proteome analysis of 5-azaC treated and drought stressed rice identifies epigenetically regulated DRGs

To identify proteins whose corresponding genes are regulated by DNA methylation and play a role in drought stress, rice seedlings were treated with 5-azaC and subjected to drought stress. Varying concentration of 5-azaC were tested and 10 µM 5-azaC was selected because concentrations of >20 µM drastically reduced the growth of rice seedlings. Two-dimensional gel electrophoresis analysis of total protein extract identified 201, 411, 205 and 501 differentially expressed spots with a fold change value of ≥2 in the control (C), control with drought stress (DS), 5-azaC (A), and 5-azaC and drought stress (ADS) samples, respectively when compared with each of the other three samples. Out of these, we analyzed 75 spots chosen based on both high fold change and resolution for precise spot elution from the gel and identified 56 proteins (Table 4) which include transcription factors, kinases/phosphatases, signaling, metabolic, and structural proteins. Except eight spots which were differentially expressed between samples C and DS, the other 48 spots (86%) were differentially expressed in relation to samples treated with 5-azaC. GO analysis of these 48 spots identified 9 genes to be involved in stress response and 5 genes in protein modification processes (Fig. S2). We identified 35 proteins that are differentially expressed (25 upregulated and 10 downregulated) in sample ADS when compared against the other three samples. Comparison of ADS against DS revealed 11 upregulated and 3 downregulated proteins (Table 4).

**Table 4:** Differentially expressed protein spots found in 5-azaC and drought treated samples.

Spot I.D	MSUv7 ID	MSU Gene Product Name	Fold change*				Cluster	Coverage	Mascot score
			C	DS	A	ADS			
C-17	LOC_Os03g38840	retrotransposon, putative, centromere-specific			2.86		-	40.20%	43.9
C-19	LOC_Os04g16830	DNA-directed RNA polymerase subunit beta, putative			9		-	18.70%	53.9
C-212	LOC_Os07g22720	2-oxo acid dehydrogenases acyltransferase domain containing protein		4.2			**-**	13.20%	84.2
C-220	LOC_Os08g38210	transcription factor BIM2, putative		3.14			EU	10.40%	64
C-241	LOC_Os08g19680	expressed protein		3.3			-	52.90%	45.6
C-260	LOC_Os03g40830	OsSub30 - Putative Subtilisin homologue, expressed		4.8			ED	6.00%	71
C-266	LOC_Os08g39840	lipoxygenase, chloroplast precursor, putative, expressed		3.91			EU	43.90%	48.3
C-615	LOC_Os10g35412	retrotransposon protein, putative, unclassified, expressed				3.03	**-**	10.70%	58.3
C-99	LOC_Os09g32670	UDP-glucuronate 4-epimerase, putative		4.25			**-**	21.00%	58.6
DS-107	LOC_Os10g21190	expressed protein				4.9	**-**	73.00%	60.5
DS-109	LOC_Os11g15570	Ser/Thr protein phosphatase family protein, putative			4.55		NED	11.20%	64
DS-14	LOC_Os03g39010	possible lysine decarboxylase domain containing protein, expressed			34.72		-	25.40%	68.6
DS-187	LOC_Os11g10480	dehydrogenase, putative				5.15	EU	13.70%	62.5
DS-19	LOC_Os03g10510	outer mitochondrial membrane porin, putative				2.41	ED	18.60%	66.7
DS-206	LOC_Os01g07120	AP2 domain containing protein, expressed	3.26				EU	14.90%	51.4
DS-278	LOC_Os03g07700	expressed protein	3.05				NEU	10.40%	65
DS-32	LOC_Os08g41620	ubiquitin carboxyl-terminal hydrolase family protein, expressed			5.37		-	36.50%	72
DS-36	LOC_Os09g25270	hypothetical protein			5.62		-	22.30%	71.5
DS-41	LOC_Os12g37400	MCM7 - Putative minichromosome maintenance MCM complex subunit 7			5.6		NED	41.30%	51
DS-64	LOC_Os12g39830	cyclin, putative				5.05	ED	23.60%	107
DS-81	LOC_Os02g41800	auxin response factor, putative			5.55		-	16.30%	99.3
A-108	LOC_Os08g39150	expressed protein		4.55			EU	14.50%	70
A-133	LOC_Os01g31220	expressed protein				5.91	-	14.20%	77.9
A-164	LOC_Os03g62290	expressed protein		6.36			-	30.90%	66
A-21	LOC_Os01g48874	wax synthase, putative		22.85			ED	18.70%	65
A-23	LOC_Os03g06540	retrotransposon protein, putative, unclassified, expressed		6.33			-	18.40%	62.2
A-234	LOC_Os09g04440	DNA-binding protein, putative				6.33	ED	13.20%	67
A-516	LOC_Os04g38810	formin, putative, expressed				2.04	ED	6.20%	71
A-676	LOC_Os12g10670	AAA-type ATPase family protein, putative				4.8	NED	26.20%	42.8
A-730	LOC_Os12g13780	retrotransposon protein, putative, Ty1-copia subclass, expressed				3.27	-	41.30%	56.3
A-93	LOC_Os11g09070	expressed protein		2.5			-	17.80%	60.6
ADS-144	LOC_Os04g44224	brain acid soluble protein 1, putative		3.93			-	29.40%	65
ADS-188	LOC_Os09g37670	expressed protein		2.21			-	56.00%	53.9
ADS-198	LOC_Os10g33800	lactate/malate dehydrogenase, putative		38.94			ED	28.30%	77.9
ADS-20	LOC_Os01g36600	PPR repeat domain containing protein, putative		6.86			ED	76.20%	47.9
ADS-212	LOC_Os04g40950	glyceraldehyde-3-phosphate dehydrogenase, putative		2.37			-	33.50%	131
ADS-292	LOC_Os07g14270	calreticulin precursor protein, putative		2.25			ED	33.50%	123
ADS-373	LOC_Os11g47760	DnaK family protein, putative		5.26			EU	13.70%	131
ADS-393	LOC_Os01g66730	exosome complex exonuclease RRP40, putative			9.51		-	23.40%	82
ADS-484	LOC_Os02g45950	expressed protein	30.82				-	15.70%	87
ADS-503	LOC_Os10g21268	ribulose bisphosphate carboxylase large chain precursor, putative	7.87	25.21			-	36.60%	179
ADS-546	LOC_Os03g08170	protein kinase APK1B, chloroplast precursor, putative	2.2				ED	70.90%	64.2
ADS-549	LOC_Os04g18660	retrotransposon protein, putative, Ty3-gypsy subclass, expressed	5.26				-	9.30%	69.4
ADS-574	LOC_Os03g16610	laccase precursor protein, putative	5.21				EU	9.80%	64
ADS-578	LOC_Os12g44170	pentatricopeptide, putative	2.02				-	6.90%	86
ADS-687	LOC_Os09g38710	HEAT repeat family protein, putative			3.37		-	42.50%	56.3
ADS-695	LOC_Os05g44720	retrotransposon protein, putative, unclassified, expressed			3.31		-	55.90%	61.9
ADS-699	LOC_Os11g47970	AAA-type ATPase family protein, putative	3.98				ED	41.40%	125
ADS-701	LOC_Os07g47230	TKL_IRAK_DUF26-lh.10 - DUF26 kinases have homology to DUF26 containing loci	16.4	12.55			-	9.00%	70.6
ADS-712	LOC_Os04g52000	protein phosphatase 2C, putative			4.51		NEU	18.10%	74
ADS-725	LOC_Os09g39180	RNA recognition motif containing protein, putative			2.27		NED	23.90%	76.5
ADS-74	LOC_Os12g19381	ribulose bisphosphate carboxylase small chain, chloroplast precursor, putative			3.45		ED	84.40%	71
ADS-742	LOC_Os08g08060	vacuolar protein sorting-associated protein 18, putative			5.05		-	5.30%	80
ADS-748	LOC_Os01g54080	kinesin motor protein-related, putative			3.11		ED	10.90%	75.9
ADS-81	LOC_Os01g69030	sucrose-phosphate synthase, putative		2.42			ED	8.30%	90.7
ADS-87	LOC_Os10g38580	glutathione S-transferase, putative		24.45			ED	22.20%	77

* denotes the fold change (upregulation) value in column Spot ID compared to the samples in sub-columns C, DS, A and ADS.

Out of the 56 identified proteins, 28 (50%) were part of DRGs considered in our cluster analysis. Nine out of the 25 proteins upregulated in the sample ADS were part of cluster ED, 5 out of which were upregulated in comparison to the sample DS. Among the five genes, the gene coding for lactate/malate dehydrogenase had a mCIP-read mapped to its promoter and the other four coding for pentatricopeptide repeat (PPR) domain containing protein, calreticulin precursor protein, sucrose-phosphate synthase and glutathione S-transferase, respectively were targets of one or more miRNAs. We also found the genes coding for DnaK (Hsp70) family protein and laccase precursor protein which are part of cluster EU to be overexpressed in ADS. The above findings suggest that these genes known to be up or downregulated under drought stress were upregulated in ADS due to the deregulation of their own methylation state or genes regulating them.

Similarly, out of the 10 genes upregulated in sample A, five were part of DRGs in cluster analysis. Out of the five genes, four were part of cluster DD and were upregulated compared to in DS or ADS samples. LOC_Os01g48874 (cluster ED) coding for wax synthase was upregulated in sample A compared to the sample DS. LOC_Os01g48874 had a mCIP-read mapped to its promoter suggesting probable activation of this gene due to demethylation. Out of the 9 spots upregulated in sample C, 3 were part of DRGs and all three were upregulated in comparison to sample DS. While the gene LOC_Os03g40830 was part of cluster ED, LOC_Os08g38210 and LOC_Os08g39840 coding for transcription factor BIM2 and LOX9, respectively were part of cluster EU.

## Discussion

The workflow pipeline of integrating genome wide epigenetic and miRNA data over DRGs, clustering and characterizing the subsets of genes with different types of molecular features revealed a number of novel insights about the key stress responsive regulatory modules. Our comparative analysis of clusters of DRGs under epigenetic/miRNA control and DRGs that are not under epigenetic/miRNA control identified a comprehensive list of molecular mechanisms and pathways (Tables S4, S5, S6, S7, S8) that are unique to each cluster. Our study generated a searchable database of DRGs with epigenetic and miRNA data (Table S1) and identified key DRGs based on connectivity with other DRGs and functional significance within sub clusters (Fig. 6A and Tables 1 and S5).

Statistically significant enrichment of features like DNA methylation reads in genic region, miRNA target sequences in DRGs compared to a random set of genes suggests DRGs are under tight epigenetic control. The negative z-score of DRGs with only DNA methylation reads in promoter or genic regions compared to random set (Fig. 2C) suggests co-occurrence of these regulatory features with other epigenetic features and can act as one of the metrics to determine the significance of a DRG based on how tightly it is regulated. In our analysis, we found a number of DRG subsets showing striking enrichment of certain features. For instance, 75% of the DRGs annotated to be involved in chromatin remodeling were downregulated. This set of genes can be further explored in determining the fitness of a drought responsive phenotype. Another interesting set of genes are the 1761 DRGs which are 32% of all DRGs considered in this study but targeted by 67% of all known/predicted miRNAs in rice which include many transcription factors targeted by more than five miRNAs, while the random set had only 12% of genes that are miRNA targets.

We found a number of DRGs with meager annotation that might be playing an important role in drought response. There are 989 DRGs (18% of all DRGs) with gene description as ‘expressed protein’ or ‘hypothetical protein’. Out of these, 806 genes (15% of all DRGs) do not have any GO annotation mappings which revealed that there are a significant number of genes involved in drought response in rice whose function is not known. In our analysis, presence of these genes in clusters associated with specific biological processes offer clues about their functional role. Further, epigenetic/miRNA features of these DRGs provide ways to manipulate their gene expression which could aid in determining their functions and also possibly identify new drought related mechanisms. For example LOC_Os03g15033 is annotated as an expressed protein with domain DUF3353. This gene is downregulated in drought stress (cluster ED) and is targeted by the highest number of miRNA (20 miRNAs that are part of miRBase).

Our results reveal the key control switches and global scale regulatory dynamics that can be potentially engineered to further enhance the process of drought adaptation. Some of the DRGs belonging to cluster EU have genes that have been well characterized including some that have shown improvement in transgenic drought adaptation (Table 5). These DRGs code for ABA-dependent signaling transduction pathway proteins, dehydrins, LEA proteins, seed storage/lipid transfer proteins, transcription factors, protein kinases, cell membrane stability-related proteins and phosphatases which increased grain yield, polyamines and osmolyte synthesis, decreased cuticular permeability and reduced water loss. Overexpression of two genes (LOC_Os11g03370 and LOC_Os01g66120) which are part of the cluster NEU code for NAC transcription factors, showed improvement in drought tolerance in transgenic studies [42], [43].

**Table 5:** DRGs in cluster EU that showed improvement in drought tolerance in transgenic studies.

Gene	Common name	Gene description	Epigenetic/miRNA features*	Reference
LOC_Os06g10880	OsABF2	bZIP transcription factor	chr6_solexa13_1001253 (g)	[45]
LOC_Os02g08230	OsGL1-2	WAX2	chr2_solexa13_1000885 (g)	[46]
LOC_Os02g50350	OsDHODH1	dihydroorotate dihydrogenase protein	osa-miRf10310-akr	[47]
LOC_Os11g29870	OsWRKY72	WRKY72	chr11_solexa14_1005447 (g);osa-miRf10273-akr;osa-miRf10576-akr	[48]
LOC_Os06g04070	OsAdc1	pyridoxal-dependent decarboxylase protein	chr6_solexa13_1000396 (p);osa-miR1848; osa-miR815a;osa-miR815b; osa-miR815c	[49]
LOC_Os02g12310	OsDREB1A	no apical meristem protein	chr2_solexa13_1001389 (g)	[50]
LOC_Os01g58420	AP37	AP2 domain containing protein	chr1_solexa12_1009761 (p)	[51]
LOC_Os02g43970	ARAG1	AP2 domain containing protein	chr2_solexa13_1007628 (p)	[52]
LOC_Os02g52780	OsbZIP23	bZIP transcription factor	chr2_solexa13_1008914 (g)	[53]
LOC_Os05g46480	OsLEA3-1	late embryogenesis abundant protein, group 3	osa-miRf11013-akr	[54]

* g and p in brackets denote that the methylation read(s) overlap genic and promoter regions respectively.

Different molecular features that we analyzed for the leaf clusters are summarized in Table 6. Overall the cluster EU seems to be made up of DRGs that mediate drought tolerance mechanisms such as osmotic adjustments, antioxidant activities and desiccation tolerance [44]. This is evident by the GOs that are unique to the cluster including protein modification and signal transduction processes (Table S4), high average fold change of gene expression (Fig. 2D), high number of TFs (Table 2), less number of PPIs (Fig. 5), enrichment of protein domains including PP2C, zf-C3H4, raffinose_syn, methyltransf_29 (Table S7) and pathways related to synthesis of amino acids, peptides and sugars which are osmoprotectants, antioxidants, protein and membrane stabilizers (Table S8). On the other hand, the cluster ED seems to be made up of DRGs that mediate processes related to drought resistance such as earliness to drought response, reduced leaf area, leaf rolling, reduced tillering, stomatal closure, efficient roots and reduced transpiration [44]. This is evident by highest number of unique GO terms (73%) including nucleosome and cytoskeletal reorganization, metabolic processes, lowest average fold change of gene expression, low number of TFs but significant enrichment of MADS-box TFs that control flowering genes among others, high number of PPIs, enrichment of p450, helicase and LRR_1 domains and enrichment of a number of biosynthetic pathways resulting in cellular adjustments and energy conservation.

**Table 6:** Comparision of different molecular features found in the leaf clusters EU, ED, NEU and NED.

	EU	ED	NEU	NED
**Average of absolute fold change**	12	3.06	20.16	3.09
**mCIP-reads in promoter region** *	280 (18.6%)	398 (20%)	0	0
**mCIP-reads in genic region**	913 (60%)	1249 (63%)	0	0
**PMRD miRNA targets**	771 (51%)	990 (50%)	0	0
**miRBase miRNA targets**	163 (10.8%)	229 (11.5%)	0	0
**ChromDB annotated genes**	22 (25%)	66 (75%)	0	0
**Unique GO terms among leaf clusters** ∧	22 (48%)	96 (73%)	9 (25.7%)	23 (49%)
**Genes with PPIs within the cluster (String-DB)**	296 (19.6%)	697 (35%)	129 (14.5%)	389 (35%)
**TF genes (PlnTFDB)**	158 (10.5%)	144 (7.2%)	103 (11.6%)	45 (4%)
**Pfam domain containing genes**	1254 (63.4%)	1617 (81.8%)	648 (73%)	829 (75%)
**Metabolic pathways (RiceCyc) unique to the cluster among the leaf clusters**	35 (18%)	28 (14%)	11 (9%)	14 (11%)
**Genes found in 5-azaC drought study among the identified protein spots** §	7 (12%)	15 (25.8%)	2 (3%)	4 (6.8%)

* percentage is no. of genes with the feature over total no. of genes in the cluster;

∧ percentage is over total no. of GO terms found in the cluster;

§ percentage over total identified protein spots.

The proteomic analysis of rice seedlings subjected to partial demethylation and drought stress was performed to test the overall effect of epigenetic mechanisms on DRGs, specifically to evaluate if there is a reversal in the differential expression of the DRGs as a result of demethylation of the promoter or gene sequence. Among the 28 proteins that matched the DRGs of our cluster analysis, there are 15 ED and 7 EU genes. Eight out of the 15 genes in cluster ED have methylation sequences in their genic or promoter regions and are upregulated in samples C and A compared to those subjected to drought stress (DS and ADS). The reversal in the expression of these genes is likely due to demethylation effect of 5-azaC. LOC_Os10g33800 coding for lactate/malate dehydrogenase has a methylation read mapped to its promoter and is highly overexpressed (39-fold) in sample ADS compared to DS. Similarly, LOC_Os01g48874 coding for wax synthase has four methylation reads mapped to its genic region and is highly overexpressed (23-fold) in sample A compared to DS. The differential expression of several other genes in sample ADS with no methylation reads in their promoter or genic regions is likely due their regulation by other genes whose methylation state was altered by 5-azaC treatment. Functional analysis of the differentially expressed genes identified by proteomic analysis would unravel the role of epigenetic regulation in drought stress response in rice.

Although many of the DRGs are extensively annotated and our analysis revealed key regulatory switches for the DRGs based on current status quo on epigenetic and miRNA mediated regulation, we expect comprehensive annotation (including siRNA, chromatin modifications and possibly other mechanisms yet to be discovered) of all the DRGs would enrich or deplete some of the striking patterns found in the clusters based on different molecular features. Thus, our study represents a first step towards the understanding of global regulatory control of stress response through integration of multiple annotation resources and unraveling a number of subsets of genes involved in key regulatory modules which could be further explored.

## Conclusions

Our analysis of DRGs as clusters based on epigenetic and miRNA features dissected biological processes and molecular functions that play a key role in the regulation of stress response. We found a number of subsets of genes showing significant enrichment of certain characteristics suggesting that these set of genes can be further studied to explore their role as regulatory modules in drought response. Understanding the influence of these regulatory modules on transcriptional/post-transcriptional gene silencing/activation and long term stress memory would be critical in engineering a drought sensitive plant variety with desirable traits into a drought resistant variety.

## Supporting Information

Figure S1 **Part of hierarchical graph tree of GO terms (AgriGO) of cluster NEU showing a number of regulatory processes at the highest level of significance (red) compared to the background reference.**
(TIF)

Figure S2 **Analysis of the GO BP terms found in the genes coding for the 48 differentially expressed protein spots in relation to the 5-azaC treatment samples.**
(TIF)

Table S1 **List of 5468 drought stress-responsive genes.**
(XLSX)

Table S2 A: List of DRGs targeted by 1 or more miRNAs in PMRD. B: List of miRNAs targeting 1 or more DRGs.
(XLSX)

Table S3 **List of 73 GO terms enriched only in one of the nine clusters.**
(XLSX)

Table S4 **Unique and overlapping GO terms in clusters NED, NEU, ED and EU.**
(XLSX)

Table S5 **List of protein-protein interactions (PPIs) shown by DRGs in cluster EU.**
(XLSX)

Table S6 **Number of different transcription factor family genes in the clusters PlnTFDB, D, NED, NEU, ED and EU.**
(XLSX)

Table S7 **Distribution of pFAM domain families in the nine clusters.**
(XLSX)

Table S8 **Metabolic pathways (RiceCyc) enriched in the clusters DU, DD, E, ED, NE and NEU.**
(XLSX)

Table S9 **Distribution of DRGs in metabolic pathways among the nine clusters.**
(XLSX)
