# Supplementary figures and images for: Bioinformatic Analysis of Epigenetic and MicroRNA Mediated Regulation of Drought Responsive Genes in Rice
Source: PLoS One. 2012 Nov 8;7(11):e49331. doi: 10.1371/journal.pone.0049331 (PMC3493535; doi:10.1371/journal.pone.0049331)

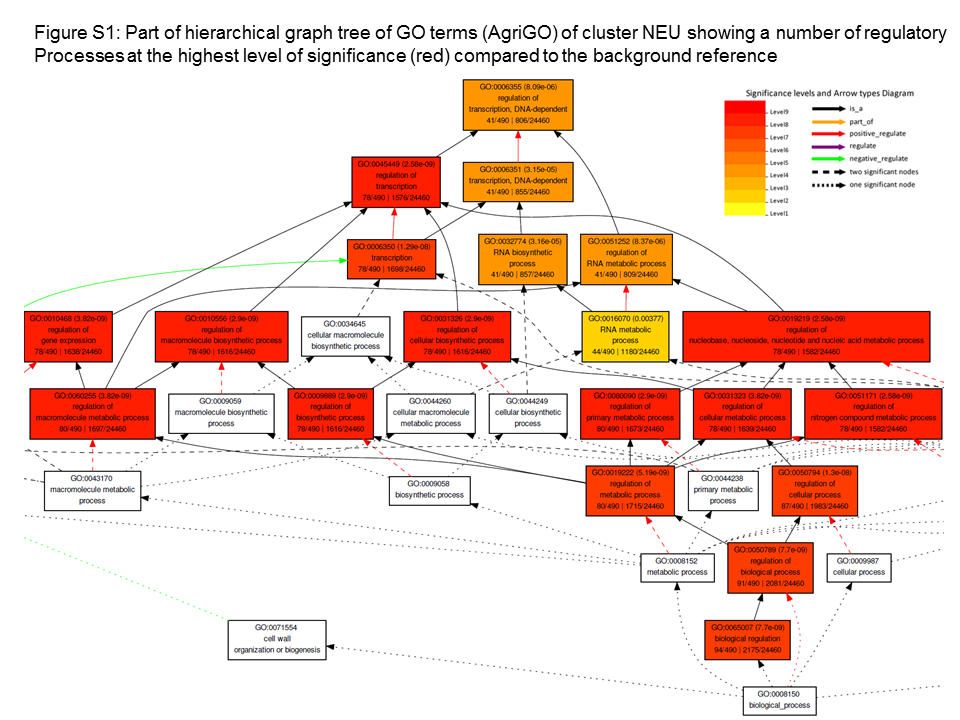

Supplement: Figure S1 — Part of hierarchical graph tree of GO terms (AgriGO) of cluster NEU showing a number of regulatory processes at the highest level of significance (red) compared to the background reference. (TIF) [file pone.0049331.s001.tif]

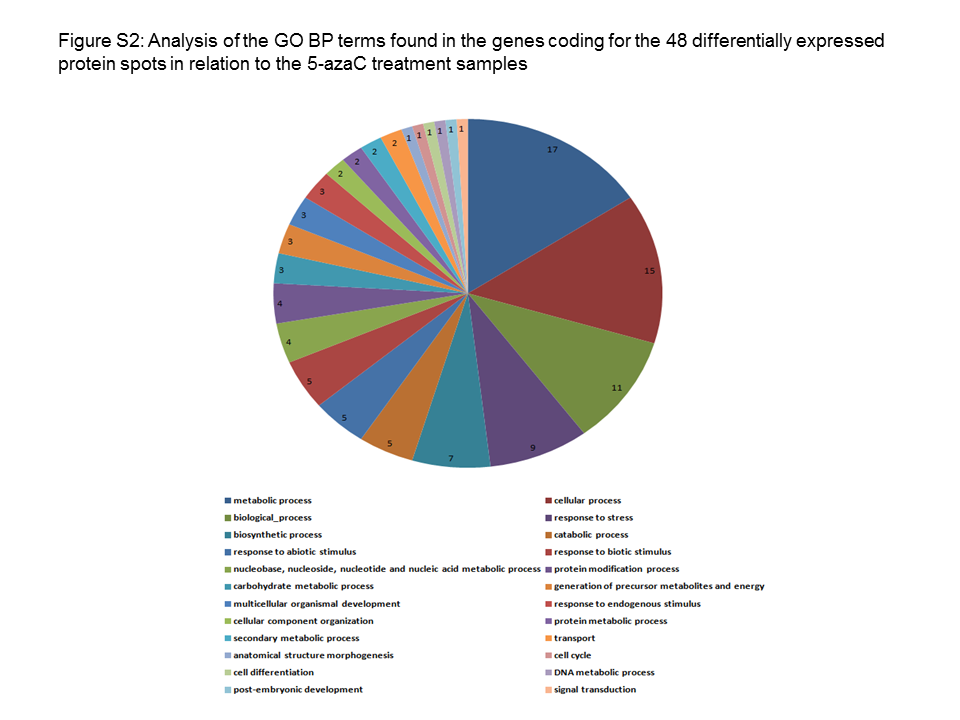

Supplement: Figure S2 — Analysis of the GO BP terms found in the genes coding for the 48 differentially expressed protein spots in relation to the 5-azaC treatment samples. (TIF) [file pone.0049331.s002.tif]
